# Comparative life tables of the potato tuberworm, *Phthorimaea operculella* , on leaves and tubers of different potato cultivars

**DOI:** 10.1093/jis/14.1.42

**Published:** 2014-01-01

**Authors:** Ali Golizadeh, Nader Esmaeili, Jabraeil Razmjou, Hooshang Rafiee-Dastjerdi

**Affiliations:** Department of Plant Protection, Faculty of Agriculture, University of Mohaghegh Ardabili, P.O.Box 179, Ardabil,Iran

**Keywords:** nutrition quality, population growth parameters, resistant cultivar, secondary metabolites, *Solanum tuberosum*

## Abstract

The potato tuberworm, *Phthorimaea operculella* Zeller (Lepidoptera: Gelechiidae), is a serious pest of the potato, *Solanum tuberosum* L. (Solanales: Solanaceae), in both fields and stores in tropical and subtropical regions. In the present study, the susceptibility of different potato cultivars to *P. operculella* was evaluated by measuring life table parameters. Tests were undertaken with leaves and tubers of 10 potato cultivars in the laboratory: Agria, Agata, Almera, Arinda, Baneba, Fiana, Marfona, Ramus, Satina, and Volvox. All parameters showed significant differences among tested cultivars. The longest mean generation times were observed on Marfona and Satina cultivars in the experiments on potato leaves and tubers, respectively. The lowest reproductive rate was observed on leaves and tubers of Marfona cultivar. Correspondingly, the lowest values of intrinsic rate of increase and the lowest finite rate of increase were also obtained on Marfona cultivar in tests on potato leaves and tubers. The highest intrinsic rate of incrase values were observed on Arinda and Baneba in the tests on leaves and tubers, respectively. The intrinsic rates of increase were significantly higher on potato leaves than on potato tubers. The lower performance of *P. operculella* on Marfona cultivar indicated that this cultivar is relatively less susceptible this pest and could be used in integrated pest management programs of *P. operculella* .

## Introduction

Cultivated potato, *Solanum tuberosum* L. (Solanales: Solanaceae), is one of the most important vegetable crops for human nutrition worldwide ( Flanders et al. 1999 ). The potato tuberworm, *Phthorimaea operculella* Zeller (Lepidoptera: Gelechiidae), is one of the most destructive insect pests of potato in tropical and subtropical potato-production regions ( Radcliffe 1982 ; Fenemore 1988 ; Visser 2005 ; Malakar and Tingey 2006 ). This pest causes damage in both field and storage ( Westedt et al. 1998 ). Female adult moths deposit eggs directly on potato foliage and tuber, and larvae cause defoliation by mining mesophyll layers in the leaves or by tunneling through tubers. Damage occurs principally in store-rooms, where previously-infested tubers engender continuous generations of *P. operculella* and damage uninfected potatoes in storage. In the field, larvae feed on both the potato foliage and the tuber, reducing the quality of production and increasing the risk of pathogen infection ( Trivedi and Rajagopal 1992 ; Ferro and Boiteau 1993 ; Sporleder et al. 2008 ; Rondon 2010 ).

The most common control method for *P. operculella* is the use of various synthetic pesticides ( Dillard et al. 1993 ). The development of resistance to insecticides and their detrimental effects on nontarget organisms has caused a growing interest in the development of alternative control methods ( Henderson and Horne 1996 ). Resistant potato cultivars could increase the efficacy of cultural and biological methods and reduce the use of insecticides ( Arnone et al. 1998 ). Host plant resistance studies have shown that there are some resistance mechanisms in the foliage and tubers of the potato cultivars ( Brown 2007 ). Musmeci et al. (1997) reported some foliar resistance on wild potatoes and interspecific hybrids.

Das et al. (1993) observed that tuber nutritional quality is an important resistance factor limiting normal growth and development of *P. operculella* larvae. Malakar and Tingey (1999) have demonstrated that foliage of the potato species *Solanum berthaultii* Hawkes (Solanales: Solanaceae) and its hybrids with the cultivated potato are resistant to oviposition by *P. operculella* , and larvae on this host plant had higher mortality and slower feeding rates than those of larvae reared on foliage of cultivated potatoes.

Host plant resistance has been used effectively in sustainable integrated management programs for several crop pests ( Dent 2000 ). Use of resistant host plants could lead to reduction in pesticide concentrations without appreciable increases in the pest population ( van Emden 1991 ). Host plants with antibiosis mechanisms can cause reduction in survival rate, size or weight, adult longevity, and fecundity of pests. Moreover, resistant host plants may have an indirect effect on pest survival by increasing the pest’s exposure to its natural enemies as a result of prolonged developmental time ( Dent 2000 ; Sarfraz et al. 2007 ).

The construction and analysis of life tables are important tools in measuring population growth capacity and understanding the dynamics of a species population under specified conditions ( Southwood and Henderson 2000 ). In applied entomology, age-specific or cohort life tables are most commonly used and the estimated parameters, particularly the intrinsic rate of population increase ( *rm* ), are the most important parameters that may be used to evaluate the level of plant resistance to insects. Host plants with lower values of *rm* are relatively more resistant than the plants with higher values of *rm* ( Razmjou et al. 2006 ).

Potato species with antixenotic resistance have been reported in some studies ( Valencia 1984 ; Malakar and Tingey 1999 , 2000), but research on life table parameters as antibiosis indices is rare. The results of the current study can provide complementary knowledge on resistant potato cultivars and could be useful in integrated pest management of *P. operculella* . Therefore, the present research provides novel information about the life table parameters of *P. operculella* on leaves and tubers of 10 potato cultivars. Knowledge of life table parameters of *P. operculella* and resistance potential of potato cultivars can serve as an important tool in planning a comprehensive program for *P. operculella* in potato fields and storehouses throughout the world.

## Materials and Methods

### Stock cultures

The initial population of *P. operculella* was provided from available infested potatoes in the laboratory of the Department of Plant Protection, University of Mohaghegh Ardabili, Iran. The stock culture of *P. operculella* was initiated on potato tubers and maintained in a breeding cage at 25 ± 1°C, 65 ± 5% RH, and a photoperiod of 14:10 L:D. The breeding cage consisted of a clear cylindrical cage (40 cm in diameter and 80 cm in height) covered by fine mesh gauze with two zips in its top and side. The adults in the stock culture were fed using a cotton piece soaked with a 10% honey solution in water. Tubers of the 10 potato cultivars (Agria, Agata, Almira, Arinda, Baneba, Fiana, Marfona, Ramus, Satina and Volvox) were provided by the Agricultural Research Institutes of Ardabil and Aligoodarz, Iran, and were planted in the research field of University of Mohaghegh Ardabili in Ardabil Province, Iran, in 2010. These varieties, especially Agria and Marfona, are commonly-grown potatoes in Iran. The leaves and tubers of potato cultivars were used for feeding *P. operculella* larvae during the experiments. Before experiments began, the population of *P. operculella* was reared for at least two generations on leaves and tubers of each potato cultivar separately. Approximately 50 male-female pairs of the newly-emerged moths from the primary colony were transferred into translucent cubic Plexiglas containers (30 ×20 ×10 cm) and were reared on each cultivar.

To obtain *P* . *operculella* eggs of the same age, 15–20 male-female pairs of the newly-emerged moths were kept inside oviposition containers. The oviposition container consisted of a clear cylindrical Plexiglas container (15 cm in diameter and 20 cm in height) covered with a fine mesh net. A filter paper on the net provided an oviposition site for the moths. The moths laid eggs on the lower surface of the filter paper. After 10–12 hr, the filter paper was removed and eggs were used in the experiments.

### Development and mortality

Development time and survival of the egg stage of *P* . *operculella* were estimated using at least 200 eggs on cultivar leaves and tubers, separately. All experiments were carried out in a growth chamber set at 25 ± 1°C, 65 ± 5% RH, and a photoperiod of 14:10 L:D. To determine the development time and survival of eggs on each potato cultivar, sections of filter paper containing 40–50 eggs each were cut off, placed in Petri dishes (10 cm diameter), and maintained in the previously-described conditions. Lids of Petri dishes were cut off and covered with fine mesh gauze for ventilation. Petri dishes were checked and the numbers of hatched eggs were recorded daily. Checking of eggs continued until all eggs hatched or collapsed. To evaluate the development of larvae on leaves and tubers of each potato cultivar, each newly-hatched larva was transferred to a clear plastic cup (15 cm diameter, 8 cm height) containing a piece of potato tuber or leaf and sand as a pupation medium. A fine hairbrush was used to transfer younger larvae on leaves or tubers. Cup lids were cut off and covered with fine mesh gauze for ventilation. At least 50 larvae were monitored on leaves and tubers of each cultivar. In the experiment on plant leaves, the petioles of detached leaves were placed into water-soaked cotton to maintain foliar freshness. The leaves were checked and replaced with fresh ones whenever necessary. Larvae normally leave tubers before pupation. All cups were checked daily, and development of larvae, pupated individuals, and their survival were recorded until adult emergence.

### Reproduction and life table parameters

For studying *P. operculella* reproduction on leaves and tubers of each potato cultivar, 13– 26 male-female pairs of the newly-emerged adult moths were used. Each pair was placed in a clear plastic cup (15 cm diameter, 8 cm height) covered with fine mesh gauze. Filter paper was placed on top of the fine mesh gauze to provide an oviposition site. A slice of fresh tuber or leaves from a potato cultivar was put on the filter paper for possible oviposition simulation. The number of eggs laid on filter paper was recorded daily and filter papers were replaced after each egg count. In addition, the number of eggs laid on the inside of cups was recorded, and those eggs were removed daily. To this end, the male and female moths were placed in a new cup while the number of eggs was recorded. Daily monitoring continued until the death of the adults. Adults were not fed during the experiments. Life table parameters including net reproductive rate ( *R _0_* ), intrinsic rate of increase ( *r _m_* ), mean generation time ( *T* ), doubling time ( *DT* ), and finite rate of increase ( *λ* ) were calculated using age-specific survival rates ( *l _x_* ) and fecundity ( *m _x_* ).

### Data analysis

Differences in *r _m_* , *R _0_* , *T* , *DT* , and *λ* values were tested for significance using the Jackknife procedure ( Maia et al. 2000 ). The steps for the application of jackknife method are described here, using *R _0_* as an example. In the first step, the value of *R _0_* was estimated by considering the survival and reproduction data for *n* females, which was referred to as true calculation ( *R _0(all)_* ). In the next step, this procedure was repeated *n* times, each time excluding a different female, therefore data of *n* – 1 females were used, and the calculated parameter was named *R _0(i)_* . The pseudovalues were calculated for each parameter for *n* samples using the following equation:

$psvR_{0(i)} = nR_{0(all)} - (n - 1)R_{0(i)}$

After calculating the *n* pseudovalues for *R ^0^* , the jackknife estimate of the mean [ *R _0( *mean* )_* ] and standard error [SEM *R _0( *mean* )_* ] were calculated by equations discussed in Maia et al. (2000) . The mean values of ( *n* – 1) jackknife pseudovalues on leaves and tubers of 10 potato cultivars were analyzed with a oneway ANOVA. If significant differences were detected, multiple comparisons were made using the Student-Newman-Keuls method ( *P* < 0.05). Statistical analysis was performed using SPSS statistical software version 16.0 (IBM, www.ibm.com ). The possible difference between leaves and tubers of each potato cultivar was analyzed via a *t* -test ( *P* < 0.05). The relationship between *r _m_* , *R _0_* , and *T* was investigated using a linear regression model.

## Results

### Immature survival rate and adult fecundity

**Figure 1.**
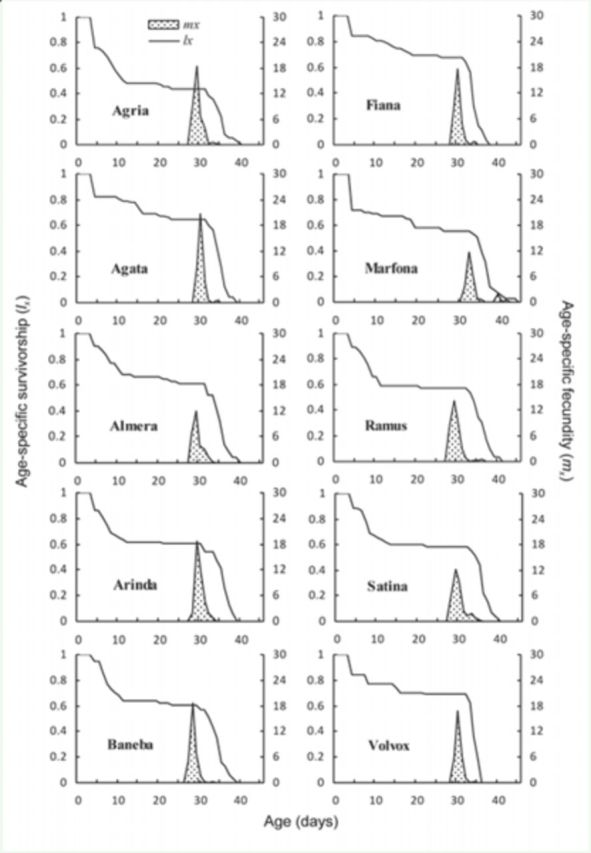
Age-specific survival rate ( *l _x_* ) and fecundity ( *m _x_* ) of *Phthorimaea operculella* reared on different potato cultivar leaves. High quality figures are available online.

**Figure 2.**
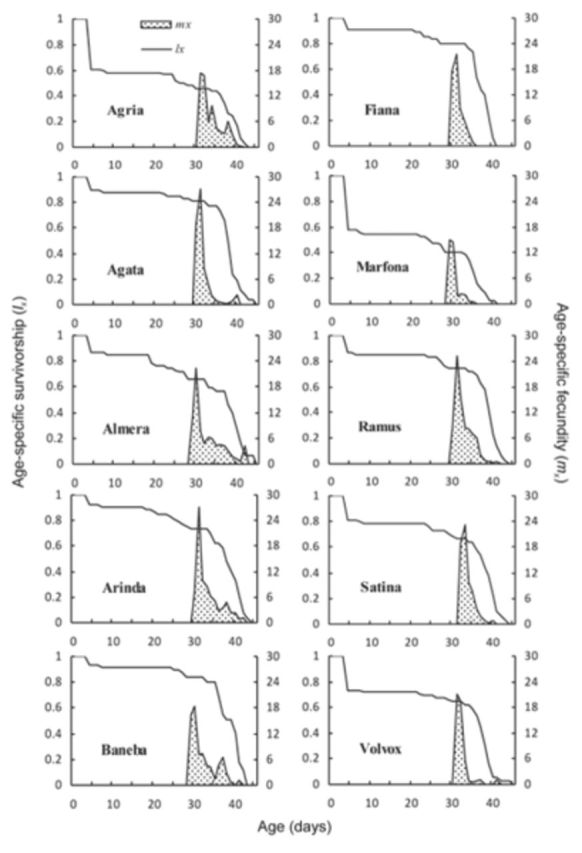
Age-specific survival rate ( *l _x_* ) and fecundity ( *m _x_* ) of *Phthorimaea operculella* reared on different potato cultivar tubers. High quality figures are available online.

Age-specific survival rates ( *l _x_* ) of *P. operculella* on leaves and tubers of different potato cultivars are shown in Figures 1 and 2 . The highest survival rates of egg, larval, and pupal stages when feeding on host plant leaves were observed on Almera, Agata, and Volvox cultivars, respectively. In the experiment on potato tubers, the highest survival rates of egg, larval, and pupal stages were observed on Baneba, Volvox, and Agata cultivars, respectively. The highest and lowest entire survival rates of individuals that developed to adulthood from the initial cohort stage were 68.9% on Volvox and 44.1% on Agria, respectively, when larvae were feeding on leaves. In the experiment on tubers, the highest and lowest entire survival rates were observed on Baneba (84.2%) and Marfona (40.6%), respectively. Among the different potato cultivars, the highest and lowest survival rates of entire development time were observed on Volvox and Agria leaves, and on Baneba and Marfona tubers, respectively. The survivorship rates on leaves were lower than those of tubers of the corresponding potato cultivar.

The age-specific fecundity ( *m _x_* ) of *P. operculella* on leaves and tubers of different potato cultivars are presented in Figures 1 and 2 . The first oviposition occurred at the ages of 28, 29, 28, 28, 27, 31, 28, 28, and 29 days of female lifetime on potato leaves of Agria, Agata, Almera, Arinda, Baneba, Fiana, Marfona, Ramus, Satina, and Volvox cultivars, respectively, and at 31, 30, 29, 30, 29, 30, 29, 30, 32, and 31 days of female lifetime on potato tubers of the same respective cultivars. The highest daily fecundity ( *m _x_* ) of *P. operculella* female adults on potato cultivars was 18.5, 20.7, 12.0, 18.6, 18.6, 17.6, 11.8, 14.2, 12.2, and 16.8 female offspring per female per day on leaves and 17.4, 27.1, 22.3, 27.2, 18.3, 21.6, 15.1, 25.2, 23.3, and 21.0 female offspring per female per day on tubers.

### Life table parameters

Life table parameters of *P. operculella* on leaves and tubers of 10 potato cultivars are given in Tables 1 and 2 . All life table parameters were affected by type of cultivar in experiments on potato leaves and tubers. The intrinsic rate of natural increase ( *r _m_* ) significantly varied in the experiments on potato leaves ( *F* = 7.099; df = 9,168; *P* < 0.01). Similarly, this parameter was significantly affected by type of cultivar when *P. operculella* was developed on potato tubers ( *F* = 19.243; df = 9,205; *P* < 0.01). The highest and lowest values of *r _m_* were on Baneba on Marfona cultivar tubers, respectively. The net reproduction rate ( *R _0_* ) was found to be significantly different between potato leaves ( *F* = 3.764; df = 9,168; *P* < 0.01) and tubers ( *F* = 19.239; df = 9,205; *P* < 0.01) depending on the potato cultivars on which individuals were developed. The *R _0_* value on leaves was lowest on Marfona cultivar and highest on Arinda. The R *0* value on tubers was lowest on Marfona cultivar and highest on Ramus.

**Table 1.**
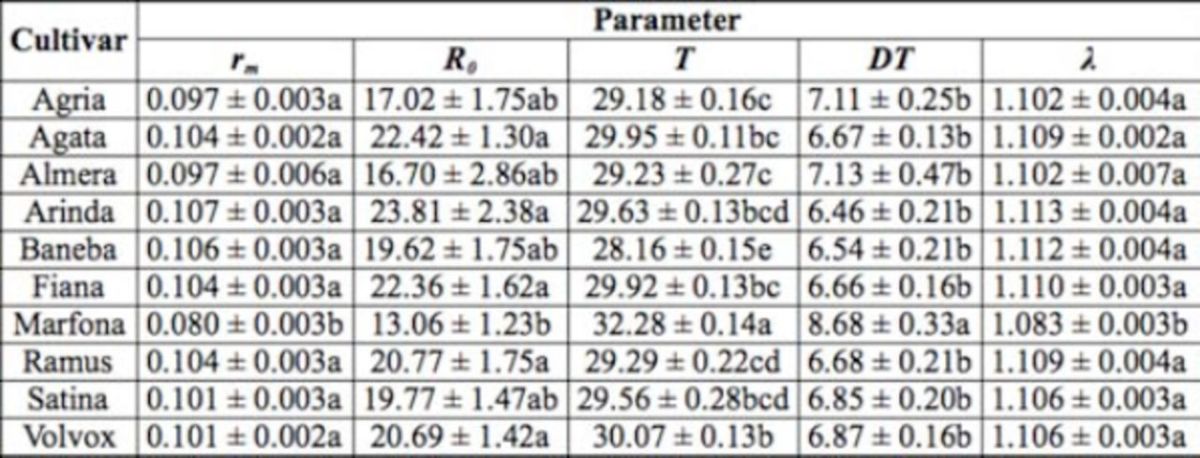
Population growth parameters (mean ± SE) of *Phthorimaea operculella* on 10 potato cultivar leaves. Means followed by the same letters within a column are not significantly different ( *P* < 0.05; Student- Newman-Keuls method after oneway ANOVA).

**Table 2.**
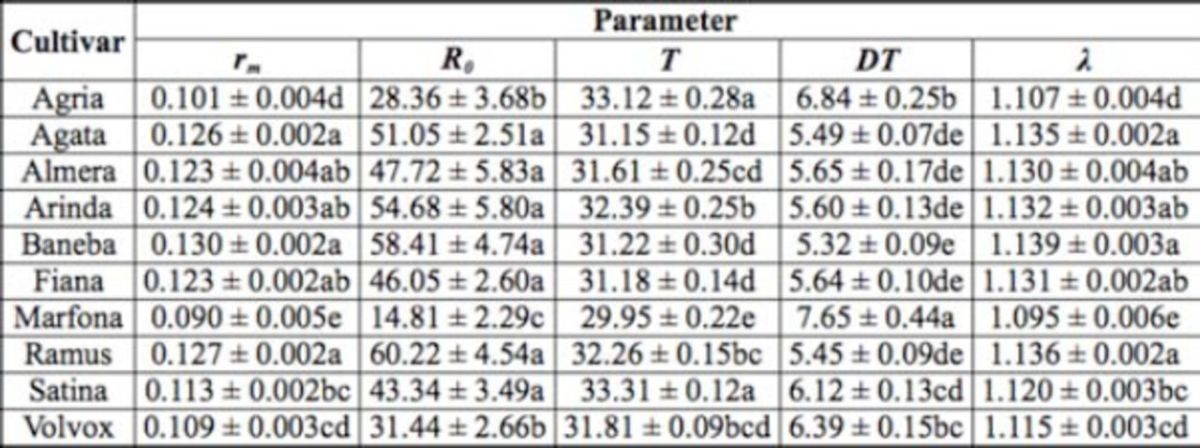
Population growth parameters (mean ± SE) of *Phthorimaea operculella* on 10 potato cultivar tubers. Means followed by the same letters within a column are not significantly different ( *P* < 0.05; Student- Newman-Keuls method after oneway ANOVA).

The mean generation time ( *T* ) of *P. operculella* was significantly affected by type of cultivar when feeding on leaves ( *F* = 45.449; df = 9,168; *P* < 0.01) and tubers ( *F* = 12.345; df = 9,205; *P* < 0.01). Mean generation time ( *T* ) values were lowest on Banbea and highest on Marfona on potato leaves, and lowest on Marfona and highest on Satina on potato tubers. The doubling time ( *DT* ) of *P. operculella* was also affected significantly by type of cultivar on leaves ( *F* = 8.469; df = 9,168; *P* < 0.01) and tubers ( *F* = 23.808; df = 9,205; *P* < 0.01). The highest and lowest *DT* values were obtained on Marfona and Arinda, respectively, on leaves, and on Marfona and Baneba, respectively, on tubers. The finite rate of increase ( *λ* ) of *P. operculella* was significantly different among potato cultivars in experiments on leaves ( *F* = 7.02; df = 9,168; *P* < 0.01) and tubers (F = 16.876; df = 9,205; P < 0.01). This parameter value was lowest on Marfona and highest on Arinda on potato leaves, and lowest on Marfona and highest on Baneba on potato tubers. All life table parameters were affected by potato tissues, and val-values on leaves were significantly higher than those on tubers ( *t-* test, *P* < 0.01).

**Figure 3.**
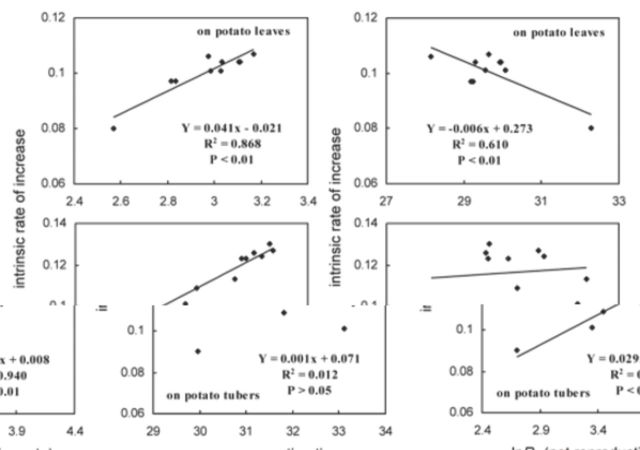
Linear regression between net reproduction rate (ln *R _0_* ), mean generation time ( *T* ) (x-axis), and intrinsic rate of increase ( *rm)* (yaxis) of *Phthorimaea operculella* . High quality figures are available online

The intrinsic rate of increase ( *r _m_* ) of *P. operculella* was significantly affected by *R _0_* (F = 52.410; df = 9; *P* < 0.01; R²= 0.610) and *T* ( *F* = 12.491; df = 9; *P* < 0.01; R²= 0.965) when feeding on potato leaves. In the experiments on tubers, the relationship between *r _m_* and *R _0_* was significant ( *F* = 124.123; df = 9; *P* < 0.01; R²= 0.940), but no significant relationship was observed between *r _m_* and *T* ( *F* = 0.099; df = 9; *P* > 0.05; R² = 0.012) ( Figure 3 ).

## Discussion

In the current study, *P. operculella* completed its lifespan on leaves and tubers of all potato cultivars successfully, but its biological traits were significantly affected by potato tissues and cultivars. For *P. operculella* , few biological studies on potato cultivars have been published ( Das et al. 1993 ; Malakar and Tingey 1999 ; Malakar and Tingey 2006 ; Rondon et al. 2009), and to our knowledge, life table parameters of this insect on leaves of different potato cultivars has never been previously reported.

The population growth parameters of *P. operculella* varied significantly on different potato cultivars in this study, which may be due to differences in plant quality and presence of nutritional factors or secondary metabolites. Plant species differ greatly in suitability as hosts for specific insects when measured in terms of survival, development, and reproductive rates ( van Lenteren and Noldus 1990 ). *Phthorimaea operculella* showed a lower performance on both leaves and tubers of Marfona cultivar, which is evident in *R _0_* values. The net reproduction rate ( *R _0_* ) is a key statistic in population dynamics ( Richard 1961 ) that summarizes the physiological traits of an insect related to its reproduction capacity. The net reproduction value of *P. operculella* on tuber of Marfona cultivar was the lowest of all cultivars. Moreover, in the experiments on host plant leaves, the lowest *R _0_* value was observed on Marfona and was significantly lower than on Agata, Arinda, Fiana, Ramus, and Volvox cultivars. Golizadeh and Razmjou (2010) studied the life table parameters of *P. operculella* on tubers of different potato cultivars, and no significant difference in *R _0_* rate among tested cultivars was reported in their study. The net reproduction rates of *P. operculella* were reported as 44.33 and 44.88 eggs on tubers of Marfona and Agria cultivars, respectively ( Golizadeh and Razmjou 2010 ), 86.18 eggs on tuber of Spunta potato cultivar ( Roux and Baumgartner 1995 ), and 69.70 eggs ( Chi 1988 ), which are much higher than the calculated values in the present study. The differences between these studies could be attributed to differences in host plant cultivars and experimental conditions.

A conclusion for suitability of potato cultivars for *P. operculella* could be made by considering both *R _0_* and *T* values,which are summarized in *r _m_* . The lower value of *R _0_* on Marfona cultivar caused the *r _m_* value to be lowest on this cultivar in experiments on both leaves and tubers. A linear regression carried out between *R _0_* and *r _m_* values showed a significant positive relationship between the two parameters in experiments on leaves. The linear relationship of *T* to *r _m_* was negatively significant on the leaves of potato cultivars. The higher *r _m_* value of *P. operculella* on Arinda cultivar was due to the greater *m _x_* and relatively higher *l _x_* rates on leaves of this cultivar. Similarly, in the experiments on tubers, the *r _m_* rates were significantly affected by R0 rates on potato cultivars, however the relationship between *T* and *r _m_* values was not significant. Therefore, the lower *r _m_* value on tuber of Marfona cultivar was mainly a result of the poor *m _x_* and lower *l _x_* of *P. operculella* on this cultivar. The differences in *r _m_* values among potato cultivars could be attributed to differences in host plant suitability due to varying levels of secondary metabolites and nutritive quality. The intrinsic rate of increase for *P. operculella* was estimated as 0.182 (day ^-1^ ) on Marfona cultivar, 0.189 (day-1) on Agria ( Golizadeh and Razmjou 2010 ), and 0.169 on Spunta ( Roux and Baumgartner 1995 ). The physiological and biochemical differences of the host plant cultivars, genetic differences resulting from laboratory rearing or variation in geographic populations of the pest, and differences in experimental conditions could be possible reasons for differences between studies ( Morgan et al. 2001 ; Liu et al. 2004 ; Yasar and Gungor 2005 ).

The present research demonstrated significant differences in life table parameters of *P. operculella* between leaves and tubers of potato cultivars. All life table parameters on leaves were significantly higher than those on tubers, which could be attributed to differences in potato tissue quality, particularly in terms of concentrations of nutritive and secondary metabolites in different parts of potato plants. Varying levels of resistance to insects occur naturally in host plant cultivars ( Stoner 1996 ). In *Solanum* spp. there are high levels of secondary metabolites, glycoalkaloids, and potatoes containing glycoalkaloids possess insecticidal properties ( Tingey 1984 ). The glycoalkaloids α-chaconine and α-solanine are most common in potatoes ( Lachman et al. 2001 ). Glycoalkaloid concentration can be affected by temperature; subsequently they are present in lower concentrations in tubers and higher concentrations in leaves, stems, and other aerial parts of potato plants ( Lachman et al. 2001 ). The higher concentrations of glycoalkaloids in potato leaves could have more negative effects on *m _x_* of *P. operculella* and this could have caused *R _0_* rates on leaves of all cultivars to be lower than on corresponding tubers. The lower *R _0_* values on leaves were reflected in the lower *r _m_* values.

In conclusion, the present study indicated that there were significant differences in life table parameters of *P. operculella* among different potato cultivars. These parameters, especially *r _m_* ,,can be used as indices for host plant resistance or suitability. Among evaluated cultivars, the greatest level of antibiotic resistance, including lower *l _x_* and lower *m _x_* , as well as the lowest *r _m_* value, were observed in Marfona cultivar on both leaves and tubers. Such antibiotic effects could cause reductions in fitness of *P. operculella* ; therefore, this cultivar could be integrated with biological and chemical controls of this pest in both fields and stores. In addition, Marfona cultivar has a high potato yield and is relatively resistant to the Colorado potato beetle ( Yasar and Gungor 2005 ), so it could have special importance in integrated pest management programs.

## Abbreviations

λ: finite rate of increase
DT: doubling time
lx: survival rate
mx: fecundity
rm: intrinsic rate of increase
R0: net reproductive rate
T: mean generation time
